# Rasch analyses of the Quick Inventory of Depressive Symptomatology Self-Report in neurodegenerative and major depressive disorders

**DOI:** 10.3389/fpsyt.2023.1154519

**Published:** 2023-06-02

**Authors:** Anthony L. Vaccarino, Sandra E. Black, Susan Gilbert Evans, Benicio N. Frey, Mojib Javadi, Sidney H. Kennedy, Benjamin Lam, Raymond W. Lam, Bianca Lasalandra, Emily Martens, Mario Masellis, Roumen Milev, Sara Mitchell, Douglas P. Munoz, Alana Sparks, Richard H. Swartz, Brian Tan, Rudolf Uher, Kenneth R. Evans

**Affiliations:** ^1^Indoc Research, Toronto, ON, Canada; ^2^Dr. Sandra Black Centre for Brain Resilience and Recovery, Hurvitz Brain Sciences Program, Sunnybrook Research Institute, Toronto, ON, Canada; ^3^Department of Medicine (Neurology), Sunnybrook Health Sciences Centre, University of Toronto, Toronto, ON, Canada; ^4^Department of Psychiatry and Behavioural Neurosciences, McMaster University, Hamilton, ON, Canada; ^5^Mood Disorders Program and Women's Health Concerns Clinic, St. Joseph's Healthcare Hamilton, Hamilton, ON, Canada; ^6^Department of Psychiatry, University of Toronto, Toronto, ON, Canada; ^7^Department of Psychiatry, University of British Columbia, Vancouver, BC, Canada; ^8^Departments of Psychiatry and Psychology, Queen's University, Providence Care, Kingston, ON, Canada; ^9^Centre for Neuroscience Studies, Queen’s University, Kingston, ON, Canada; ^10^Rotman Research Institute, Baycrest Health Sciences, Toronto, ON, Canada; ^11^Department of Psychiatry, Dalhousie University, Halifax, NS, Canada

**Keywords:** neurodegenerative disorder, Rasch measurement theory, depressive symptoms, validity, QIDS-SR, major depressive disorder

## Abstract

**Background:**

Symptoms of depression are present in neurodegenerative disorders (ND). It is important that depression-related symptoms be adequately screened and monitored in persons living with ND. The Quick Inventory of Depressive Symptomatology Self-Report (QIDS-SR) is a widely-used self-report measure to assess and monitor depressive severity across different patient populations. However, the measurement properties of the QIDS-SR have not been assessed in ND.

**Aim:**

To use Rasch Measurement Theory to assess the measurement properties of the Quick Inventory of Depressive Symptomatology Self-Report (QIDS-SR) in ND and in comparison to major depressive disorder (MDD).

**Methods:**

De-identified data from the Ontario Neurodegenerative Disease Research Initiative (NCT04104373) and Canadian Biomarker Integration Network in Depression (NCT01655706) were used in the analyses. Five hundred and twenty participants with ND (Alzheimer’s disease or mild cognitive impairment, amyotrophic lateral sclerosis, cerebrovascular disease, frontotemporal dementia and Parkinson’s disease) and 117 participants with major depressive disorder (MDD) were administered the QIDS-SR. Rasch Measurement Theory was used to assess measurement properties of the QIDS-SR, including unidimensionality and item-level fit, category ordering, item targeting, person separation index and reliability and differential item functioning.

**Results:**

The QIDS-SR fit well to the Rasch model in ND and MDD, including unidimensionality, satisfactory category ordering and goodness-of-fit. Item-person measures (Wright maps) showed gaps in item difficulties, suggesting poor precision for persons falling between those severity levels. Differences between mean person and item measures in the ND cohort logits suggest that QIDS-SR items target more severe depression than experienced by the ND cohort. Some items showed differential item functioning between cohorts.

**Conclusion:**

The present study supports the use of the QIDS-SR in MDD and suggest that the QIDS-SR can be also used to screen for depressive symptoms in persons with ND. However, gaps in item targeting were noted that suggests that the QIDS-SR cannot differentiate participants falling within certain severity levels. Future studies would benefit from examination in a more severely depressed ND cohort, including those with diagnosed clinical depression.

## Introduction

Symptoms of depression are present across a broad range of neurodegenerative disorders (ND), and can negatively impact quality of life, functioning and progression of disease (1–6). Given the comorbidity of depression in ND and its relation to poorer outcomes, it is important that depression-related symptoms be adequately screened and monitored in persons living with ND. Depression in ND can be a challenge to identify, however, as some symptoms of depression overlap with the manifestation of ND-related signs and symptoms, such as difficulties in concentration, fatigue, restlessness, sleep-and appetite-related problem and feeling of being slowed down (7–10).

The Quick Inventory of Depressive Symptomatology Self-Report (QIDS-SR) (11) is a widely used self-reported, symptom-based rating scale that is aligned with DSM-IV criteria for major depressive disorder (MDD) and can therefore be a useful tool to assess and monitor depressive severity across different patient populations. The QIDS-SR was originally developed to assess depressive severity in MDD (11), and since then has been used across a broad range of diverse patient populations (12–19). However, it’s psychometric properties in ND have not been investigated.

Given the overlap of symptoms of depression and ND, it is important that the measurement properties of the QIDS-SR also be assessed in persons living with ND. In this context, we used Rasch Measurement Theory (RMT) (20–26) to assess the psychometric properties of QIDS-SR in ND. For comparison, RMT was also applied to QIDS-SR in a MDD sample, as the QIDS-SR was originally developed in that population (11). RMT considers the probability of an item’s score as a function of both the person’s individual trait level (depressive severity) and the item’s difficulty (severity level where 50% of respondents will endorse the item). RMT provides fundamental criteria for objective scale measurement and determines how well the observed data approximates the Rasch measurement model. Items that do not fit the Rasch model are indications that they are measuring more than one construct, and thus possibly confounded by the presence of ND-related signs and symptoms. Rasch-based criteria assessed included undimensionality and item-level goodness of fit, category ordering, item targeting, person separation index and reliability and item bias (differential item functioning) (22, 25, 26).

## Methods

### Study population and datasets

The present study used de-identified data collected as part of the Ontario Neurodegenerative Disease Research Initiative (ONDRI, NCT04104373) and Canadian Biomarker Integration Network in Depression (CAN-BIND trial 1, NCT01655706). These programs are part of the Ontario Brain Institute’s Integrated Discovery Programs, which supports collaborative research networks focused on various brain conditions, including neurodevelopmental disorders, cerebral palsy, epilepsy, mood disorders, and neurodegenerative diseases (27–29). As part of the overall Integrated Discovery Programs, common data elements (CDEs) are collected across all participating programs as means of supporting cross-disease research, including the demographic information and QIDS-SR assessments used in the present analyses (6).

ONDRI is a prospective multi-site research program designed to characterize and track progression of neurodegenerative and neurovascular disorders (30, 31). Five hundred and twenty male and female participants who met criteria for one of the following ND participated in the study: Alzheimer’s disease or amnestic single or multidomain mild cognitive impairment (AD/MCI, *n* = 126), amyotrophic lateral sclerosis (ALS, *n* = 40), cerebrovascular disease (CVD, *n* = 161), frontotemporal dementia (FTD, *n* = 53) and Parkinson’s disease (PD, *n* = 140) (Table 1). Please see Farhan et al. (30) and Sunderland et al. (31) for full protocol details, inclusion and exclusion criteria.

**Table 1:** Demographic and clinical characteristics.

	ND	MDD
N	520*	177
AGE, YEARS ± SD (RANGE)	68.59 ± 7.72 (40–88 years)	35.69 ± 12.52 (18–61 years)
SEX, % FEMALE	33.5%	62.3%
QIDS-SR ± SD (RANGE)	4.81 ± 3.39 (0–19)	9.59 ± 4.83 (0–24)

* ADMCI (*n* = 126), ALS (*n* = 40), CVD (*n* = 161), FTD (*n* = 53), PD (*n* = 140).

CAN-BIND is a multi-site research program designed to identify biomarkers of antidepressant response in MDD (32, 33). Two-hundred and eleven male and female outpatients between 18 and 60 years old whose symptoms met criteria for a major depressive episode in the context of MDD, according to the Diagnostic and Statistical Manual of Mental Disorders, Fourth Edition, Text Revision (DSM-IV-TR), as determined by the Mini International Neuropsychiatric Interview and had a MADRS score of ≥24, participated in the first CAN-BIND study (CAN-BIND-1). As the MADRS entry criteria in CAN-BIND-1 would in effect restrict the range of scores and skew the data toward higher scores at baseline, Rasch analyses were applied to Week 8 MDD participant data (*n* = 177) (see Table 1). Please see Lam et al. (33) for full protocol details, inclusion and exclusion criteria.

Studies were carried out in accordance with the Declaration of Helsinki and the International Council for Harmonization of Technical Requirements for Pharmaceuticals for Human Use (ICH) guidelines, and the study designs and procedures were reviewed by the appropriate ethics committees; informed consent was obtained from all participants after full explanation of the nature of the procedures.

### Assessments

Demographic and QIDS-SR items are CDEs that were collected across all OBI-funded programs to support cross-disease comparisons and were used in the present analyses (6). All assessments were captured electronically on REDCap (Research Electronic Data Capture)^1^ within the Brain-CODE informatics platform^2^ (29).

QIDS-SR is a 16-item self-report measure that assesses the severity of depressive symptoms based on DSM-5 criteria for major depressive episode, with items scored on a 4-point scale (0–3) (11). Scoring of the QIDS-SR converts the 16 items into nine DSM domains (sad mood, concentration, self-criticism, suicidal ideation, interest, energy/fatigue, sleep disturbances and changes in appetite/weight). Because of challenges in providing immediate follow-up with those expressing suicidal ideation, item #12 assessing suicidality was omitted from the ONDRI protocol and therefore removed from all analyses (8 domains with total score ranging from 0–24). This was not expected to impact the scale’s ability to discriminate depression, as removal of suicidality items in similar scales [for example, PHQ-9 (with suicide item) vs. PHQ-8 (without suicide item)] does not impact psychometric properties (34).

### Analyses

Demographic and clinical characteristics were calculated and compared across all cohorts; ANOVA was used for comparison of continuous variables (age) and chi-squared for comparison of categorical variables (sex). Analyses were performed using SPSS V27. A level of *p* < 0.05 was regarded as statistically significant. RMT was used to assess scale performance across cohorts. Rasch analyses were carried out using WinSteps Version 5.1.

#### Unidimensionality

The Rasch model assumes unidimensionality and local independence of items. Unidimensionality was examined by principal component analysis of the residuals derived from the Rasch model. The scale was considered unidimensional if >40% of variance was explained by the measurement variable and unexplained variance of the first contrast accounts for <10% or has an eigenvalue <2 (35–37). Local dependency was assessed by examining residual correlations between items (38), with correlations of *r* < 0.4 suggesting no consequential response dependency (36, 37).

#### Item fit

Item-level goodness fit statistics were calculated as an index of how much the observed score for a given item within the scale deviates from the expected score of the Rasch model (i.e., are the items measuring a single underlying construct?). Items that did not fit the model (misfits) do not contribute to measurement of the underlying construct and likely add unwanted noise to the scale. Item “infit” mean square (MNSQ) values provide a fit index for each item that are in near proximity to the person’s severity level, and “outfit” MNSQ values for differences between observed and expected values for items that are far from the person’s severity level. MNSQ values between 0.5 and 1.5 were considered acceptable fit, with values between 1.5 and 2 considered to underfit the model and do not contribute to measurement of the underlying construct but do not distort the results, and values greater than 2.0 flagged as misfits that can distort the scale (39).

#### Category threshold ordering

Category probability curves were generated to test the ordering of the response categories to determine whether participants can discriminate between the ordered response options, with fit values between 0.5 and 1.5 considered to be acceptable fit, and values greater than 2.0 flagged as misfitting (39).

#### Item targeting

Person-item maps were constructed that plot individual participants and items on a single continuum to compare the range and position of the person measure distribution to that of the item measure distribution. Both item “difficulty” (i.e., level of depressive severity that item assesses) and person “ability” (i.e., level of depressive severity) are visualized together on a logit scale (i.e., log of the odds); with the right side of the map displaying the items from most difficult (top) to least difficult (bottom) and the left side plotting the individual participants, with those at the top having the highest trait level (symptom severity) and those at the bottom the lowest. The targeting of the scale is assessed by comparing mean person and mean item logit locations, with good measurement targeting evidenced when mean persons and items difficulty are in close proximity to one another (within 1 logit) (40, 41). The clinical utility of summing individual items from a scale to form a total score of overall severity requires that the items be spread out across the severity level of a broad range of persons. Gaps between items are considered problematic, as persons falling within those gaps cannot be differentiated from one another.

#### Differential item functioning

Ideally, a scale should perform similarly regardless of the subgroup being assessed (i.e., generalizability of the scale). To assess any potential item biases, differential item functioning (DIF) was used to determine whether items have any differences in item difficulty between subgroups of interest. That is, whether subgroups with similar levels of depression have the same probability of endorsing a given item. The existence of DIF was assessed for dichotomous categories based on sex (male vs. female) and cohort (ND vs. MDD), with mean differences in person measures of >0.43 logits representing slight-to-moderate DIF and > 0.64 logits as indications of moderate-to-large DIF (with *p* < 0.05 in Rasch-Welch test statistic) (40, 41).

#### Reliability and separation index

Reliability of the QIDS-SR was evaluated using the person separation index and reliability derived from the Rasch model. The person separation index evaluates the scale in terms of its ability to distinguish participants into distinct levels of severity (strata), with person separation indices of >1.5 considered acceptable (representing a minimum required to separate sample into 2 distinct strata, i.e., low and high). Person reliability is analogous to Cronbach’s alpha as a measure of internal constancy among all scale items, with values of >0.70 as indication of acceptable model fit (39).

## Results

Demographic characteristics are shown in Table 1. The MDD cohort was predominately female (62.3%); consistent with the higher rates of depression reported by females (42–44). By contrast, the ND cohort was predominantly male (66.5%). The bias toward male participants in the ND cohort is likely related to ONDRI enrollment strategy that required presence of a spousal study partner that may have influenced sample demographics (6, 31). As expected, QIDS-SR total scores were higher in MDD than ND (9.59 ± 4.83 and 4.81 ± 3.39, respectively). Please see (31) and (32) for comprehensive demographic and clinical characteristics of ND and MDD cohorts, respectively.

### Unidimensionality

Unidimensionality of the QIDS-SR was supported by PCA with variances explained by the measurement variables of 46.4 and 57.5% and unexplained variance of the first residual of 11.1% (eigenvalue of 1.66) and 7.6% (eigenvalue of 1.42) in MDD and ND, respectively. No consequential response dependency was found, with all residual correlations <0.4.

### Item fit

Item difficulty estimates and fit statistics are shown in Table 2. Item difficulty estimates ranged from −2.83 logits (SLEEP) and 1.04 logits (SELF-PERCEPTION) for the ND cohort and from −1.47 logits (SLEEP) to 0.53 logits (PSYCHOMOTOR) in the MDD cohort. All items showed acceptable goodness-of-fit statistics in the ND cohort, with MNSQs ranging from 0.64 to 1.50. In the MD cohort, most items showed acceptable goodness-of-fit, with minor underfitting noted for APPETITE (infit MNSQ = 1.57; outfit MNSQ = 1.61) and SELF-PERCEPTION (infit MNSQ = 1.55) (See Table 2).

**Table 2:** QIDS-SR item difficulty estimates, fit statistics, and differential item functioning.

	ND			MDD			DIF
	Item difficulty logit	Infit MNSQ	Outfit MNSQ	Item difficulty logit	Infit MNSQ	Outfit MNSQ	DIF contrast** (ND vs. MDD)
SADNESS	0.68	0.99	0.95	0.09	0.63	0.71	0.44*
SLEEP	−2.83	1.09	1.27	−1.47	1.00	1.05	−0.77*
APPETITE	−0.17	1.37	1.34	0.06	1.57	1.61	−0.13
CONCENTRATION	0.26	0.88	0.90	0.10	0.65	0.65	0.19
SELF-PERCEPTION	1.04	1.50	1.02	0.33	1.55	1.42	0.55*
INTEREST	0.90	0.87	0.64	0.34	0.93	0.84	0.35
ENERGY	−0.05	0.79	0.77	0.03	0.71	0.69	0.00
PSYCHOMOTOR	0.17	0.85	0.69	0.53	0.96	1.00	−0.49*

*p < 0.05 (Rasch-Welch statistic); **QIDS-SR total scores ranging from 6–15 were used for DIF.

### Category threshold ordering

**Figure 1:**
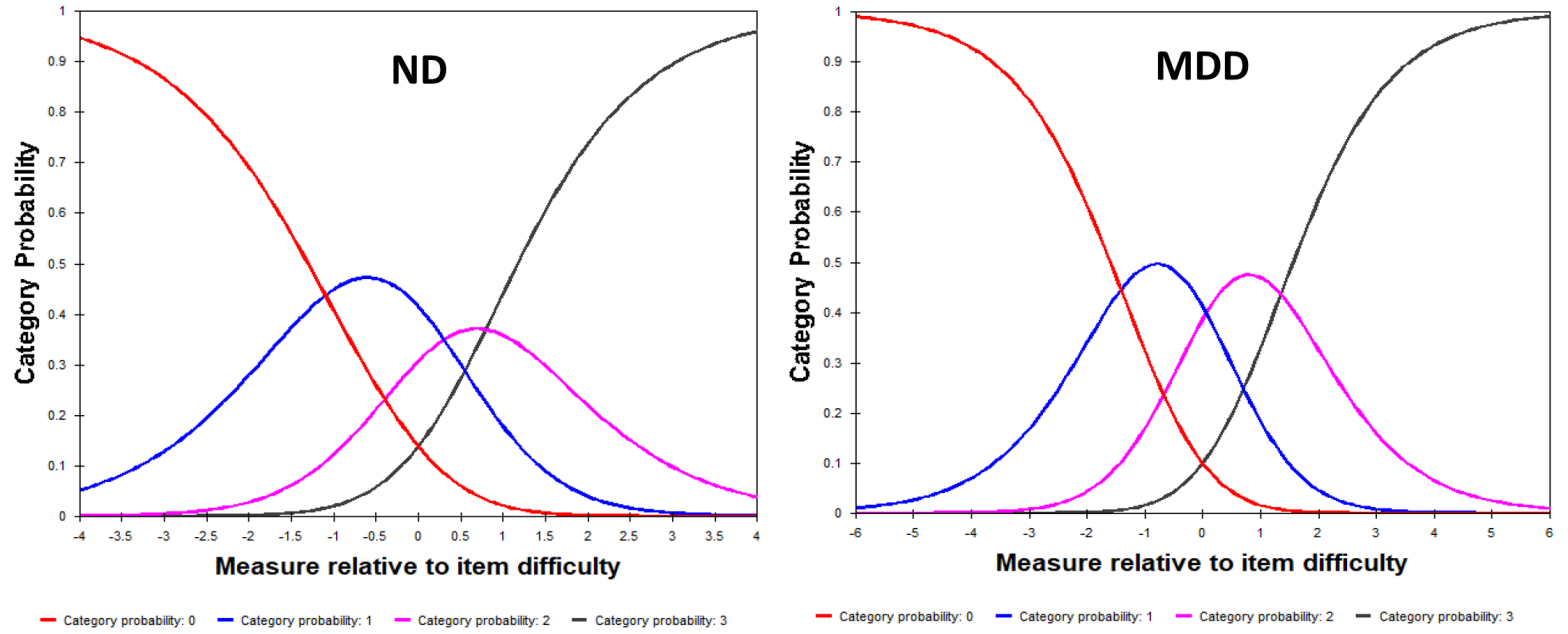
Category Probability Curves for the QIDS-SR in ND **(left panel)** and MDD **(right panel)**.

Category probability curves for the QIDS-SR showed that scale options functioned in sequential order in capturing increasing levels of severity, with participants with higher levels of depression endorsing higher QIDS-SR options for both ND and MDD cohorts (Figure 1). Underfitting (infit MNSQ = 1.71; outfit MNSQ = 1.94) was noted for the most severe level (option 3) in the ND cohort, which is likely to be related the low endorsement of this option (5%), as compared with MDD (27%).

### Item targeting

**Figure 2:**
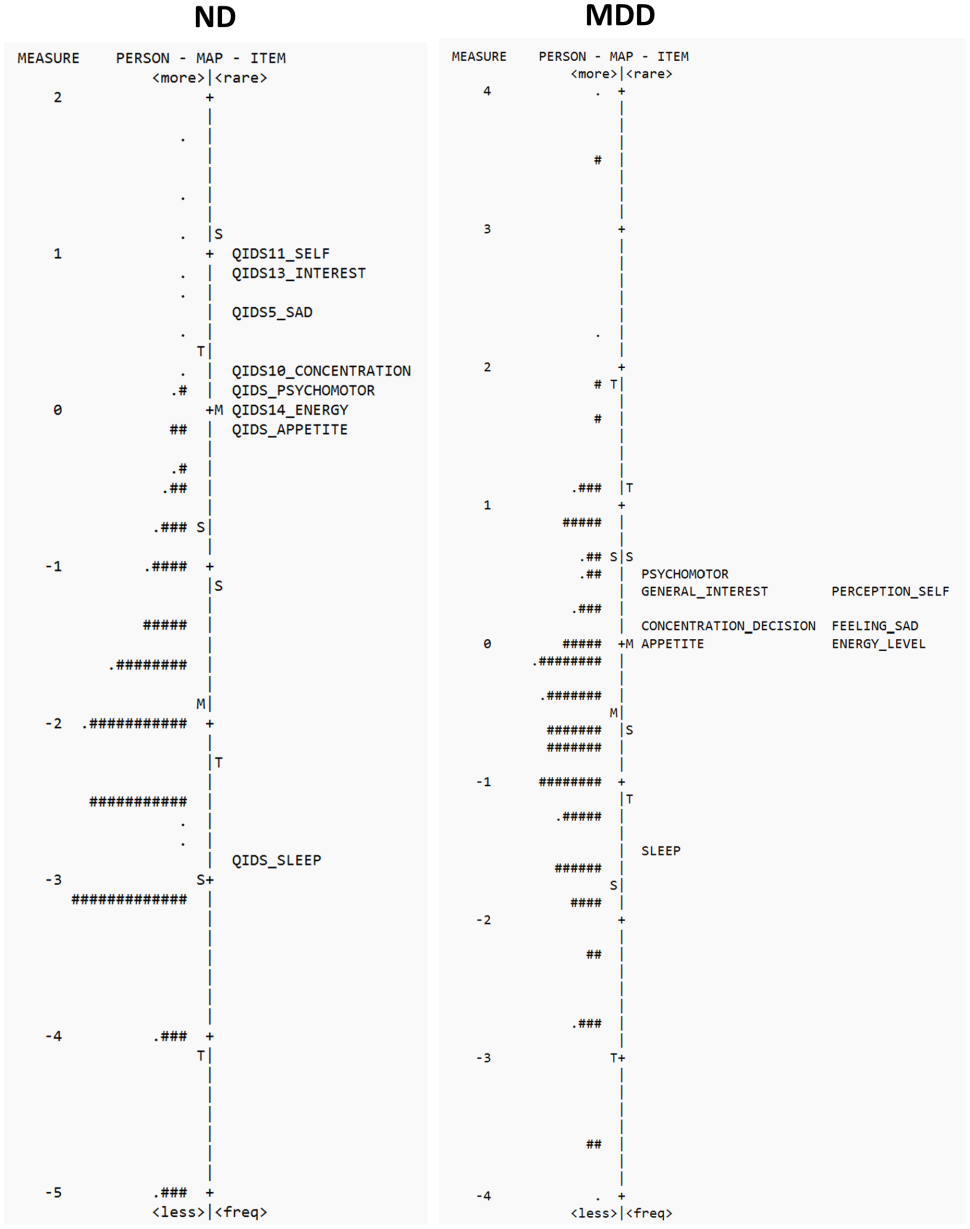
Person-item location (Wright maps) for QIDS-SR items in ND **(left panel)** and MDD **(right panel)**. The right side of the map displays the items from most difficult **(top)** to least difficult **(bottom)**, and the left side plots the individual participants, with those at the top having the highest trait level (depression) and those that the bottom the least. M, mean difficulty; S, one standard deviation; T, two standard deviations.

Person-item locations (Wright maps) for the QIDS-SR in ND and MDD are shown in Figure 2. The proximity of the mean item measure (logit = 0) to the mean person measure (logit = −1.89) in the ND cohort suggests that QIDS-SR items target a more severe depression than experienced by the ND cohort. This is not surprising given that the participants with a diagnosis of MDD were excluded from the study, as per ONDRI protocol (30). Furthermore, the gap of items between −2.83 logits and 0.17 logits suggest poor precision for persons falling between those severity levels (See Figure 2). In the MDD cohort, the proximity of the mean item measure (logit = 0) to the mean person measure (logit = 0.56) indicates satisfactory item targeting in MDD. However, further inspection of Figure 2 shows a gap of items targeting persons between −1.46 logits and 0.03 logits, suggesting poor precision for persons falling within that range of severity (see Figure 2). Please see Supplementary Table S1 for QIDS-SR raw scores and corresponding person measure logit estimates.

### Differential item functioning

As part of the ONDRI protocol, individuals with “unstable psychiatric illness defined as psychosis (hallucinations or delusions), lifelong history of major depression, or untreated late-onset major depression within 90 days of the screening visit” were excluded from the study (30). As a result, individuals with more severe psychiatric symptoms or a clinical diagnosis of MDD were excluded from the ONDRI study. Therefore, to assess DIF in ND and MDD participants within similar ranges of depression, we compared those with total QIDS scores ranging from 6–15, which represents mild-to-moderate levels of depression (MDD = 9.72 + 2.50, *n* = 114; ND = 8.54 + 2.37, *n* = 159). DIF between ND and MDD cohorts was noted for 4 of the 8 items, with SADNESS (DIF contrast = 0.44, *p* < 0.05) and SELF-PERCEPTION (DIF contrast = 0.55, *p* < 0.05) more likely to be endorsed in MDD than ND, and SLEEP (DIF contrast = −0.77, *p* < 0.05) and PSYCHOMOTOR (DIF contrast = −0.49, *p* < 0.05) more likely to be endorsed in ND than MDD (see Table 2). These differences suggest that ND participants presenting with depressive symptoms are more likely to report somatic-related problems (sleep and psychomotor) and less likely to report non-somatic symptoms of sadness and negative self-perception. This latter finding is consistent with ONDRI’s exclusion of persons with an MDD diagnosis (30); a diagnosis that requires the presence of either depressed mood or loss of interest. No DIF was noted based on sex. As this was secondary use of data the impact of additional demographic and clinical factors was not examined, and limited sample sizes precluded subgroup analyses. Future studies would benefit from a better understanding of the generalizability of these results (i.e., ND-diagnosis) and impact of potential confounding factors (i.e., medication use, comorbidities, disease severity).

### Reliability and separation index

Person separation indices and reliability were acceptable in MDD, indicating that the QIDS-SR could stratify participants into two separate groups (1.86 and 0.78, respectively). By contrast, person separation indices and reliability were low in ND (1.28 and 0.62, respectively) indicating that the scale is not sensitive enough to separate the present ND cohort into different groups.

## Discussion

The present study used retrospective data pooled across two large cohort studies in persons with ND and MDD to evaluate the measurement properties of the QIDS-SR to assess symptoms of depression. Although the psychometric properties of the QIDS-SR have been well established in MDD and other populations (12–19), to our knowledge this is the first time its psychometric properties have been assessed in persons with ND.

In the ND cohort, the QIDS-SR showed favorable psychometric properties that support its construct validity, including unidimensionality and acceptable goodness-of-fit statistics, suggesting that all items contributed to the same underlying construct of depression. With respect to item difficulty, SLEEP was the least difficult/most endorsed item and SELF-PERCEPTION the most difficult/least endorsed item. The low person separation index and reliability, however, suggest that in its present form the QIDS-SR cannot distinguish different levels of severity in the ND sample used. This is further highlighted by the person-item map showing poor targeting at lower person severity levels (see Figure 2). This is not surprising given that the QIDS-SR is based on DSM symptom domain criteria for a major depressive episode and is therefore more targeted to a clinically depressed population. Additional studies are required therefore to assess the performance of the QIDS-SR in a broader ND population that includes individuals with a clinical diagnosis of depression.

Consistent with previous literature, the results of the present analysis provide further support for the utility of the QIDS-SR in assessing symptom severity in MDD (11, 16). With respect to item difficulty, SLEEP was the least difficult/most endorsed item and PSYCHOMOTOR the most difficult/least endorsed item. However, some shortcomings of the QID-SR in assessing depression in MDD were noted. Although person-item maps demonstrated satisfactory item targeting between mean person and item measures in MDD, the lack of items between −1.46 logits and 0.03 logits suggests poor measurement precision for persons falling between those lower severity levels (See Figure 2). The precision of the QIDS-SR would benefit, therefore, with the development of new items that target those severity levels (25). Incorporating Rasch analyses will aid in the development new items that target these gaps (25, 45). In addition, determining which symptoms might be relevant should also incorporate input form those affected and then tested in broader populations (46, 47) For example, previous studies have shown assessment of symptoms such as irritability, rumination and cognitive difficulties that are not assessed in the QIDS-SR can provide additional targeting across severity levels in MDD (25, 47). It is important to also note that the QIDS item #12 that assess thoughts of suicide was omitted from the analysis; and inclusion of this item in the assessment provides additional targeting of those with higher levels of depression (25).

Underfitting was noted for the APPETITE item in MDD (see Table 2), thus calling into question the construct validity of this item and may add unwanted noise to the scale. Indeed, although changes in appetite are included in DSM-5 symptom criteria for MDD, studies have shown that appetite-related depression items show poor psychometric properties in MDD (25, 48), and appetite-related symptoms are variably expressed in MDD (49) and can be confounded by the presence of antidepressant side effects (50). It is also important to note that the QIDS-SR APPETITE item assesses both increases and decreases in appetite and weight as one item domain, whereas the MADRS, that was used to define the CAN-BIND-1 entry criteria, only assesses decreases in appetite. It is possible, therefore, that persons with these “atypical” reverse vegetative symptoms (increased appetite and weight gain) may have been underrepresented in the CAN-BIND cohort. All other items showed acceptable goodness-of-fit to the Rasch model, thus supporting their construct validity in MDD.

Person separation index and reliability (as well as Cronbach’s alpha) were satisfactory and suggest in the present MDD cohort two distinct levels of depression could be differentiated. Category probability curves were also favorable and demonstrate that QIDS-SR response option categories could be differentiated using the 4-point scale. With respect to item hierarchy and difficulty, SLEEP was considered the least difficult/most endorsed item and PSYCHOMOTOR the most difficult/least endorsed item.

DIF between ND and MDD is interesting and suggests that the presentation of depressive symptoms in ND may be more somatic in nature than MDD, with the ND cohort more likely to report PSYCHOMOTOR difficulties and problems with SLEEP, and less likely to report SADNESS and SELF-PERCEPTION (see Table 2). The reduced non-somatic symptoms in the ND cohort can be explained by ONDRI’s exclusion criteria of MDD diagnosis which would have effectively selected against non-somatic depression-related symptoms, such as sadness. It is also possible that non-motor symptoms can go underrecognized in ND, including blunted facial expression that could impact identification of mood-related symptoms (51). With respect to somatic symptoms of depression in the ND cohort, it could be argued that the assessment of depression in ND is confounded by the presence of overlapping ND-related signs and symptoms (8–10). However, the acceptable fit statistic and unidimensionality of the QIDS-SR in the ND cohort would argue against this interpretation and support it’s construct validity as a measure of depression in ND that is not confounded by the presence of ND-related signs and symptoms. Similarly, the PHQ-9, which is also based on DSM criteria for depression, has also been shown to be a valid scale in ND populations despite the presence of overlapping somatic symptoms (7, 10, 52). From a clinical perspective, these results suggest that the presentation of depression may be different between ND and MDD, and that clinicians explore somatic signs and symptoms in persons with ND as possible underlying depression.

It is important to note that age-matched controls were not included in this study, and thus we cannot rule out age-related changes in depression. Indeed, previous studies examining the relationship between age and presentation of depressive symptoms suggests that older adults with depression are more likely to report somatic symptoms of depression than younger depressed adults (53, 54). Therefore, although the differences noted in the present study cannot exclude possible age-related effects, these results none-the-less highlight the importance of recognizing somatic presentation of depression in older adults with ND. This may be particularly important in developing therapeutic interventions in persons with ND and comorbid depression, as somatic symptoms of depression are associated with poorer QoL, worse treatment outcome and treatment resistance (55, 56).

In addition to differentiating ND- and depression-related symptoms, it’s also possible that age-related and cognitive difficulties could impact the ability to understand and elicit appropriate responses from participants. Although the present study did not assess the impact of ND-related difficulties on QIDS-SR responses, previous studies have shown that reliability and validity of patient-reported outcome measures are not impacted by cognition or age in PD (57) and the QIDS-SR has been well validated in aged populations (14). Furthermore, the ONDRI inclusion criteria required a minimum MOCA score, English fluency and visual ability, as part of the ONDRI protocol (30, 31), and participants were offered the option to have the QIDS-SR administered *via* a coordinator to allow clarification. However, although the ONDRI protocol would be expected to minimize the impact of age and ND-related symptoms, additional studies are required to better understand the impact ND-related difficulties on QIDS-SR responses.

In conclusion, the results of the present study are consistent with previous reports supporting the use of the QIDS-SR in MDD (11, 16) and suggest that the QIDS-SR may also be useful to screen for depressive symptoms in persons with ND. There are caveats, however, including item targeting issues that impacts scale’s ability to differentiate across different levels of severity. In addition to providing a framework to generate validation (i.e., unidimensionality and model fit), RMT also provides empirical evidence as to where scale performance can be improved to reduce noise and increase precision (25, 45). Visual examination of person-item maps, for example, shows that the QIDS-SR lacks precision across the full spectrum of severities, suggesting that more items are needed to target those falling within those gaps (25). Additional studies would also benefit from examination in a more severely depressed ND cohort, including those with diagnosed clinical depression.

## Data availability statement

Participants’ data used in this study are currently stored in the Brain-CODE Neuroinformatics Platform (https://www.braincode.ca/) managed by the Ontario Brain Institute. Requests to access these datasets should be directed to the Ontario Brain Institute at info@braininstitute.ca.

## Ethics statement

The studies involving human participants were reviewed and approved by all recruitment sites in accordance with the Governance Policy of Ontario Brain Institute as well the institutional policies. The patients/participants provided their written informed consent to participate in this study.

## Author contributions

AV: study concept and design, statistical analysis, interpretation of data, and manuscript preparation. KE and RU: study design and interpretation of data. BF, SK, RWL, and RU: CAN-BIND recruiting site leads. SB, MM, DM, and RS: ONDRI recruiting site leads. SGE, MJ, B Lasalandra, EM, AS, and BT: data management and curation. All authors provided review of the manuscript for important intellectual content, read, and approved the final manuscript.

## Funding

This research was conducted with the support of the Ontario Brain Institute, an independent non-profit corporation, funded partially by the Ontario government. BEAM funding from Brain Canada, the Edwards Foundation and GE Healthcare for in kind support; funding was also received from Linda C. Campbell toward the BEAM study. CAN-BIND and ONDRI are Integrated Discovery Programs with support from the Ontario Brain Institute, with funding and/or in-kind support also provided by the investigators’ universities and academic institutions. Additional funding for CAN-BIND was provided by CIHR, Lundbeck, Bristol-Myers Squibb, Pfizer, and Servier. The funders were not involved in the study design, collection, analysis, interpretation of data, the writing of this article or the decision to submit it for publication.

## Conflict of interest

RM has received consulting and speaking honoraria from AbbVie, Allergan, Eisai, Janssen, KYE, Lallemand, Lundbeck, Neonmind, Otsuka, and Sunovion, and research grants from CAN-BIND, CIHR, Janssen, Lallemand, Lundbeck, Nubiyota, OBI and OMHF. RWL has received honoraria for *ad hoc* speaking or advising/consulting, or received research funds, from: Asia-Pacific Economic Cooperation, Bausch, BC Leading Edge Foundation, Brain Canada, Canadian Institutes of Health Research, Canadian Network for Mood and Anxiety Treatments, CAN-BIND Solutions, Carnot, Grand Challenges Canada, Healthy Minds Canada, Janssen, Lundbeck, Lundbeck Institute, Michael Smith Foundation for Health Research, MITACS, Myriad Neuroscience, Ontario Brain Institute, Otsuka, Pfizer/Viatris, Sanofi, Unity Health, Vancouver Coastal Health Research Institute, and VGH-UBCH Foundation. SK has received funding for Consulting or Speaking engagements from Abbvie, Boehringer-Ingelheim, Janssen, Lundbeck, Merck, Neurotorium, Otsuka, Pfizer, Sunovion, and Servier. He has received Research Support from Abbott, Brain Canada, CIHR (Canadian Institutes of Health Research), Janssen, Lundbeck, Ontario Brain Institute, Otsuka, SPOR (Canada’s Strategy for Patient-Oriented Research). He has stock/stock options in Field Trip Health.

The remaining authors declare that the research was conducted in the absence of any commercial or financial relationships that could be construed as a potential conflict of interest.

## Publisher’s note

All claims expressed in this article are solely those of the authors and do not necessarily represent those of their affiliated organizations, or those of the publisher, the editors and the reviewers. Any product that may be evaluated in this article, or claim that may be made by its manufacturer, is not guaranteed or endorsed by the publisher.

## Footnotes

^1^www.project-redcap.org

^2^www.braincode.ca
