# Supplementary material for: Rasch analyses of the Quick Inventory of Depressive Symptomatology Self-Report in neurodegenerative and major depressive disorders
Source: Front Psychiatry. 2023 Jun 2;14:1154519. doi: 10.3389/fpsyt.2023.1154519 (PMC10273843; doi:10.3389/fpsyt.2023.1154519)
Supplement: Supplementary file 1 [file Table_1.DOCX]

**Appendix: Supplementary Table 1.** QIDS-SR raw scores and corresponding person measure logit estimates.

| QIDS-SR  Raw Score | Logit Score  (ND) | Logit Score  (MDD) |
| --- | --- | --- |
| 0 | -5.40 | -4.89 |
| 1 | -4.06 | -3.61 |
| 2 | -3.14 | -2.80 |
| 3 | -2.51 | -2.29 |
| 4 | -2.03 | -1.90 |
| 5 | -1.64 | -1.58 |
| 6 | -1.32 | -1.30 |
| 7 | -1.04 | -1.05 |
| 8 | -0.79 | -0.81 |
| 9 | -0.56 | -0.59 |
| 10 | -0.34 | -0.38 |
| 11 | -0.13 | -0.17 |
| 12 | 0.07 | -0.03 |
| 13 | 0.28 | 0.24 |
| 14 | 0.49 | 0.44 |
| 15 | 0.70 | 0.65 |
| 16 | 0.92 | 0.86 |
| 17 | 1.16 | 1.08 |
| 18 | 1.42 | 1.32 |
| 19 | 1.70 | 1.59 |
| 20 | 2.02 | 1.89 |
| 21 | 2.41 | 2.25 |
| 22 | 2.92 | 2.73 |
| 23 | 3.71 | 3.49 |
| 24 | 4.98 | 4.74 |
